# HnRNPR-CCNB1/CENPF axis contributes to gastric cancer proliferation and metastasis

**DOI:** 10.18632/aging.102254

**Published:** 2019-09-16

**Authors:** Er-Bao Chen, Xuan Qin, Ke Peng, Qian Li, Cheng Tang, Yi-Chou Wei, Shan Yu, Lu Gan, Tian-Shu Liu

**Affiliations:** 1 Department of Medical Oncology, Zhongshan Hospital, Fudan University, Shanghai, China; 2 School of Chemical Biology and Biotechnology, Shenzhen Graduate School of Peking University, Shenzhen, China; 3 Center of Evidence-based Medicine, Fudan University, Shanghai, China

**Keywords:** hnRNPR, gastric cancer, RNA binding protein, CCNB1, CENPF

## Abstract

Gastric cancer (GC) is a common disease globally with high mortality rate. It is therefore necessary to develop novel therapies targeting specific events in the pathogenesis of GC. Some hnRNP family members are involved in multiple cancer biological behaviors. However, the potential function and mechanism of hnRNPR, a new molecule of hnRNP family in GC remains unknown. We found that the expression of hnRNPR was significantly overexpressed in multiple cancers compared to the normal tissues. Functionally, hnRNPR promoted cancer cell proliferation, migration, and invasion. Knockdown of hnRNPR in two type mice models, with two types of tumors models decreased the tumor aggressiveness and metastasis. Mechanistically, hnRNPR targeted oncogenic pathways by stabilizing the expression of CCNB1 and CENPF mRNA level. Knockdown of CCNB1 and CENPF abolished the hnRNPR-induced cell growth and invasion, respectively. Furthermore, the protein level of hnRNPR in the tumor was positively correlated with the expression of CCNB1 and CENPF in clinical samples. Together, these results indicate that overexpression of hnRNPR promoted the aggressiveness of GC by increasing the mRNA expression of CCNB1 and CENPF. HnRNPR-CCNB1/CENPF axis may be a potential therapeutic target for GC treatment.

## INTRODUCTION

Gastric cancer (GC) is the fifth most common cancer and the third leading cause of cancer-related deaths in the world [1]. Although great progress has been made in terms of surgical and interventional therapy in recent years, the outcome of GC patients is still not satisfactory. In many countries, the overall five-year survival of patients with GC is low (~30%) [2]. Although several potential biomarkers have been proposed to monitor GC progression and chemoresistance, such as Long Noncoding RNA GMAN [3], and estrogen-related receptor gamma [4], IRTKS [5], none has been approved for clinical use. Thus, exploring novel targets and the underlying mechanisms of GC development is urgently needed.

Growing evidence indicates that many RNA-binding proteins are possible cancer biomarkers as they regulate a series of biological processes including tumor initiation, development and drug resistance [6–8]. The heterogeneous nuclear ribonucleoproteins (hnRNPs), an RNA-binding protein, can bind to initial transcripts and are involved in all aspects of (pre)mRNA processing including gene transcription, alternative splicing, RNA stabilization, subcellular transport, and degradation control [9–12]. Further, some hnRNPs have been found to play a role in splicing, gene expression and metabolism across multiple cancer types, such as hnRNPK in cholangiocarcinoma [11], hnRNPI in colorectal cancer [13], hnRNPA1 and hnRNPAB in hepatocellular carcinoma [14, 15]. HnRNPR was originally identified as a component of the hnRNP family. It interacts with hnRNP complexes to regulate pre-mRNA and mature mRNA transcripts [16]. Recent studies have revealed that recombinant hnRNPR enhanced transcriptional activity of c-fos promoter [17]. Immunoprecipitation coupled with mass spectrometry analysis demonstrated that hnRNPR interacted with SOX2, a key transcription factor in that regulates stemness of cells [18]. MicroRNAs (miRNAs) are short, endogenous, single-stranded RNA (~22 nucleotides) that modulate gene expression and cell function. MiRNAs originate from polyA-tailed primary and precursors (60~70 nucleotides) transcripts that undergo complex and different processing steps until they achieve functional maturity [19]. During biogenesis, the binding of hnRNPR to hnRNPH1 contributes to the versatility of miRNA [20, 21]. Moreover, several potential mechanisms of hnRNPR have been reported, such as splicing, transport of RNAs and regulation of RNA stability [22]. Currently, the function of hnRNPR and the molecular mechanisms in cancer progression are not known.

In the present work, the function of hnRNPR in GC was investigated. First, we assessed whether the mRNA level of hnRNPR is overexpressed in pan-cancers, including gastric cancer. Second, based both gain-or loss-of function assays, it was found that hnRNPR is an oncogene in GC as it promotes cell proliferation by inducing cell cycle progression in vitro and in vivo assays. The oncogenic activity of hnRNPR was dependent on its ability to stabilize the mRNA of CCNB1 and CENPF, which are key mediators of cell cycle and tumor metastasis, respectively. Clinically, expression of hnRNPR was positively correlated with the expression of CCNB1 and CENFP. Collectively, these results demonstrated that hnRNPR functions as an oncogene in GC and directly controls the fate of cancer cells and their metastasis.

## RESULTS

### HnRNPR is overexpressed in human gastric cancer

**Figure 1:**
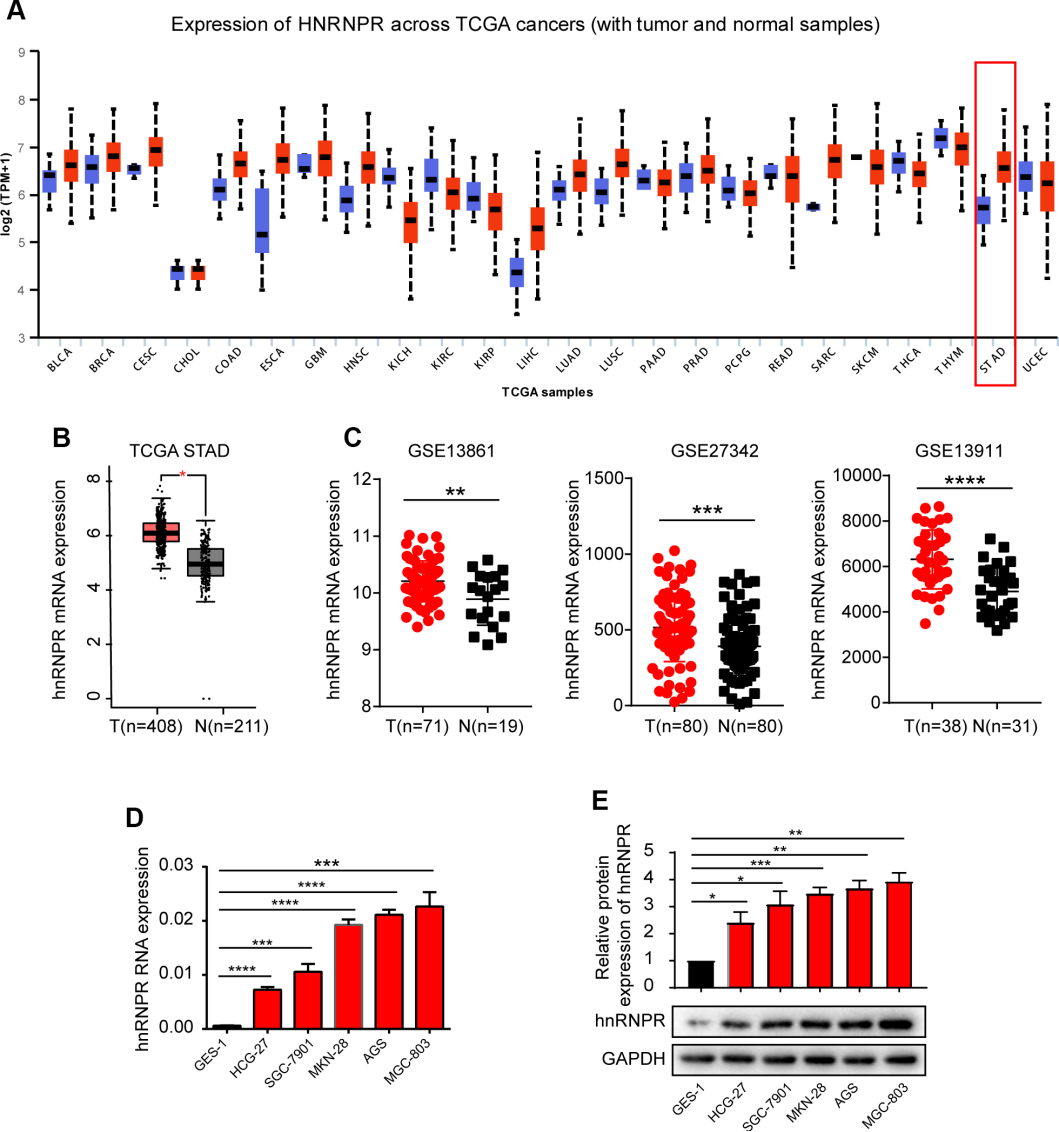
**hnRNPR was overexpressed in gastric cancer cell lines and patients.** (**A**) The bioinformatics analysis revealed that hnRNPR was upregulated in stomach tissues compared to normal tissues. (**B**) The GEPIA dataset indicated that the level of hnRNPR in cancer tissues was higher compared to that in normal tissues. T(Tumors)=408; N(Normal)=211. (**C**) Three GEO database (GSE13861, GSE27342, GSE13911 from GEO database) revealed that the expression of hnRNPR was significantly higher in tumors compared with normal tissues. (**D**) The relative mRNA level of gastric cell lines (SGC-7901, AGS, HGC-27, MKN-28, MGC-803) were elevated than in normal epithelial cell line GES-1. GAPDH served as the internal control. (**E**) The protein level of hnRNPR in gastric cell lines were higher than in normal cell lines GES-1. GAPDH served as the internal control. Each experiment was performed in triplicate and repeated three times. P values were calculated with two-tailed unpaired Student’s t test. *, P<0.05, **, P<0.01, ***, P<0.001, ****, P<0.0001 versus the control.

Although the role of many hnRNP family members in different human cancers has been reported, little is known about the role of hnRNPR in tumors, particularly in gastric cancer. To address this, the expression of hnRNPR was analyzed in pan-cancers via bioinformatics. In the UALCAN database (available at http://ualcan.path.uab.edu/cgi-bin/Pan-cancer.pl?genenam=HNRNPR), hnRNPR was upregulated in multiple cancers compared with corresponding normal tissues (Figure 1A). To further verify the statistical significance of this difference in expression, the expression of hnRNPR was analyzed in GC samples and corresponding controls in the STAD dataset (The Cancer Genome Atlas, TCGA). HnRNPR mRNA was significantly overexpressed in GC tissues compared to normal tissues (Figure 1B). Notably, hnRNPR was also upregulated in many cancers, including cholangiocarcinoma, Lymphoid neoplasm diffuse large B-cell lymphoma, Glioblastoma multiforme, bran lower grade glioma, pancreatic adenocarcinoma, and thymoma (Supplementary Figure 1). To further verify these findings in microarray datasets, the hnRNPR expression was compared between match GC and normal tissues derived from three GEO datasets. The analysis of hnRNPR expression ratio in tumor/non-tumor matched tissues revealed that the expression of hnRNPR was significantly increased in GC (Figure 1C). These findings suggested that hnRNPR may play a key role in tumor progression. The endogenous expression status of hnRNPR in five gastric cancer cell lines (SGC-7901, AGS, HGC-27, MKN-28, MGC-803) and one normal epithelial cell line GES-1 was examined. Results showed that both mRNA and protein expression were significantly increased in GC cell lines than that in normal cell line (Figure 1D, 1E). This suggested that hnRNPR is highly expressed in GC cell lines.

### HnRNPR promotes GC viability and tumor aggressiveness in vitro

**Figure 2:**
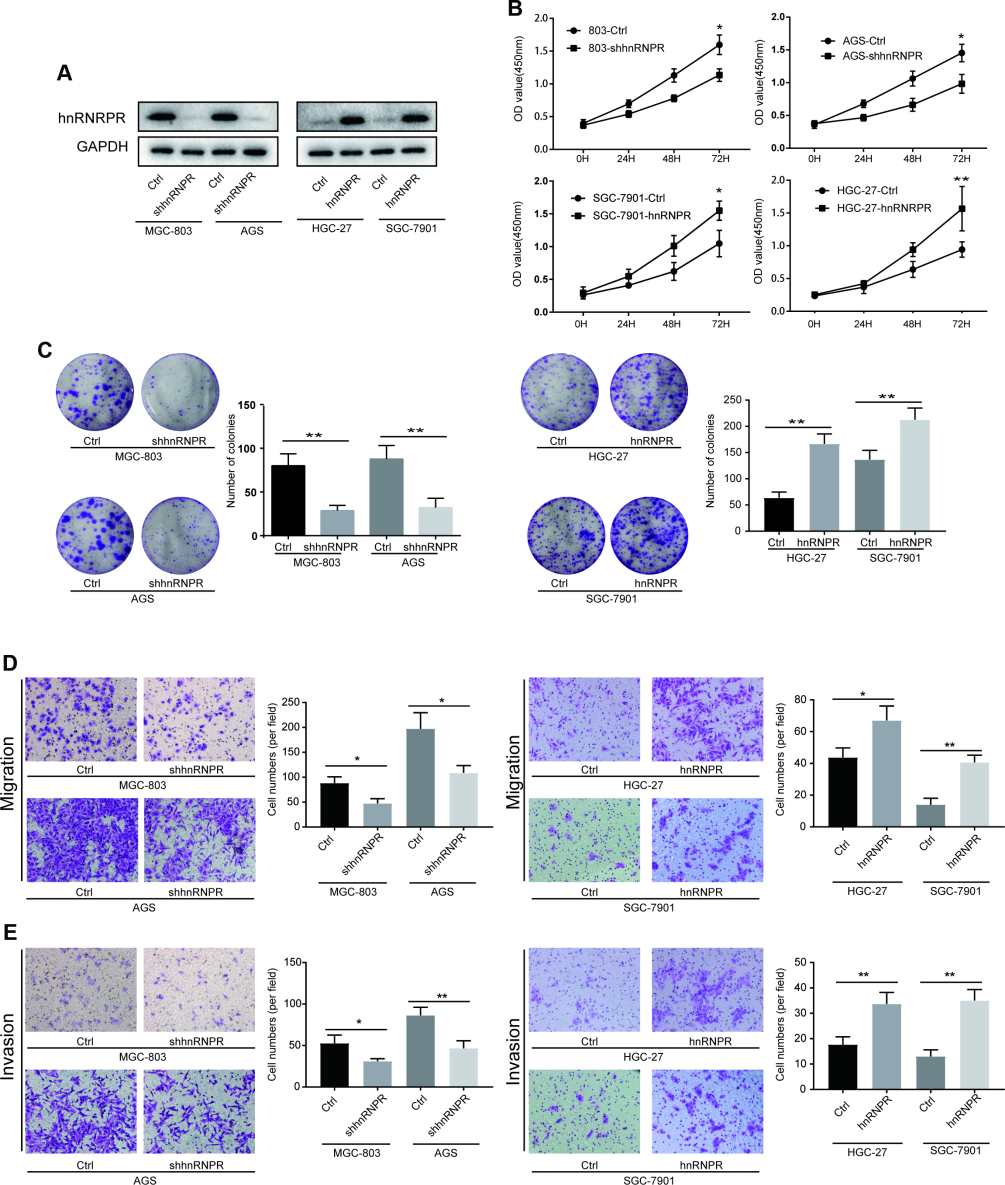
**hnRNPR promoted cell proliferation, migration and invasion of gastric cancer cells.** (**A**) The efficiency of hnRNPR knockdown or overexpression was detected by western blot in the indicated cells after transfection with shhnRNPR or plasmids. GAPDH serve as the internal control. (**B**) CCK8 and (**C**) colony formation assays showed that hnRNPR knockdown suppressed cell growth in MGC-803 and AGS, while overexpression of hnRNPR promoted cell proliferation in HGC-27 and SGC-7901. (**D**) Migration assays and (**E**) invasion assays revealed that hnRNPR inhibition decreased cell migration and invasion abilities of MGC-803 and AGS, and hnRNPR overexpression increased cell migration and invasion abilities in HGC-27 and SGC-7901. Each experiment was performed in triplicate and repeated three times. P values were calculated with two-tailed unpaired Student’s t test. *, P<0.05, **, P<0.01.

Given that hnRNPR expression was upregulated in GC, we speculated that hnRNPR might act as an oncogene in GC. To explore the molecular function of hnRNPR in the cell growth of gastric cell in vitro, hnRNPR was overexpressed in HGC-27 and SGC-7901 or knocked-down in MGC-803 and AGS using lentiviral-based approaches (Figure 2A). CCK8 and clone formation assays were carried out to detect the impact of hnRNPR on cellular growth. The CCK8 experiments showed that the cell growth rate in hnRNPR-overexpression cells was markedly higher than that of the control cells at various time points, whereas knockdown of hnRNPR significantly inhibited cell growth of MGC-803 and AGS cells compared with controls (Figure 2B). Similarly, the clone formation experiments indicated that overexpression of hnRNPR promoted clonogenicity of GC cells and inhibition of hnRNPR suppressed the number of colonies of GC cells (Figure 2C). These results demonstrated that hnRNPR promoted GC cell growth in vitro. Subsequently, to characterize the effect of hnRNPR on the metastatic ability of GC cells, Transwell experiments with or without Matrigel and wound-scratch assay were performed in GC cells. Compared with the control groups, hnRNPR overexpression remarkably enhanced the cell metastatic ability of HGC-27 and SGC-7901, whereas knockdown of hnRNPR markedly decreased the migration, invasion rate and number of MGC-803 or AGS cells compared with controls (Figure 2D, 2E). The wound-scratch assay also confirmed the conclusion that hnRNPR upregulated the migration ability of GC cells. The ectopic hnRNPR overexpression in cells promoted healing, whereas knockdown of hnRNPR in cells delayed wound healing (Supplementary Figure 2A). Thus, these results indicated that hnRNPR promoted cell proliferation and invasion of GC.

### HnRNPR potentiates CCNB1 stability at mRNA level to facilitate cell growth

**Figure 3:**
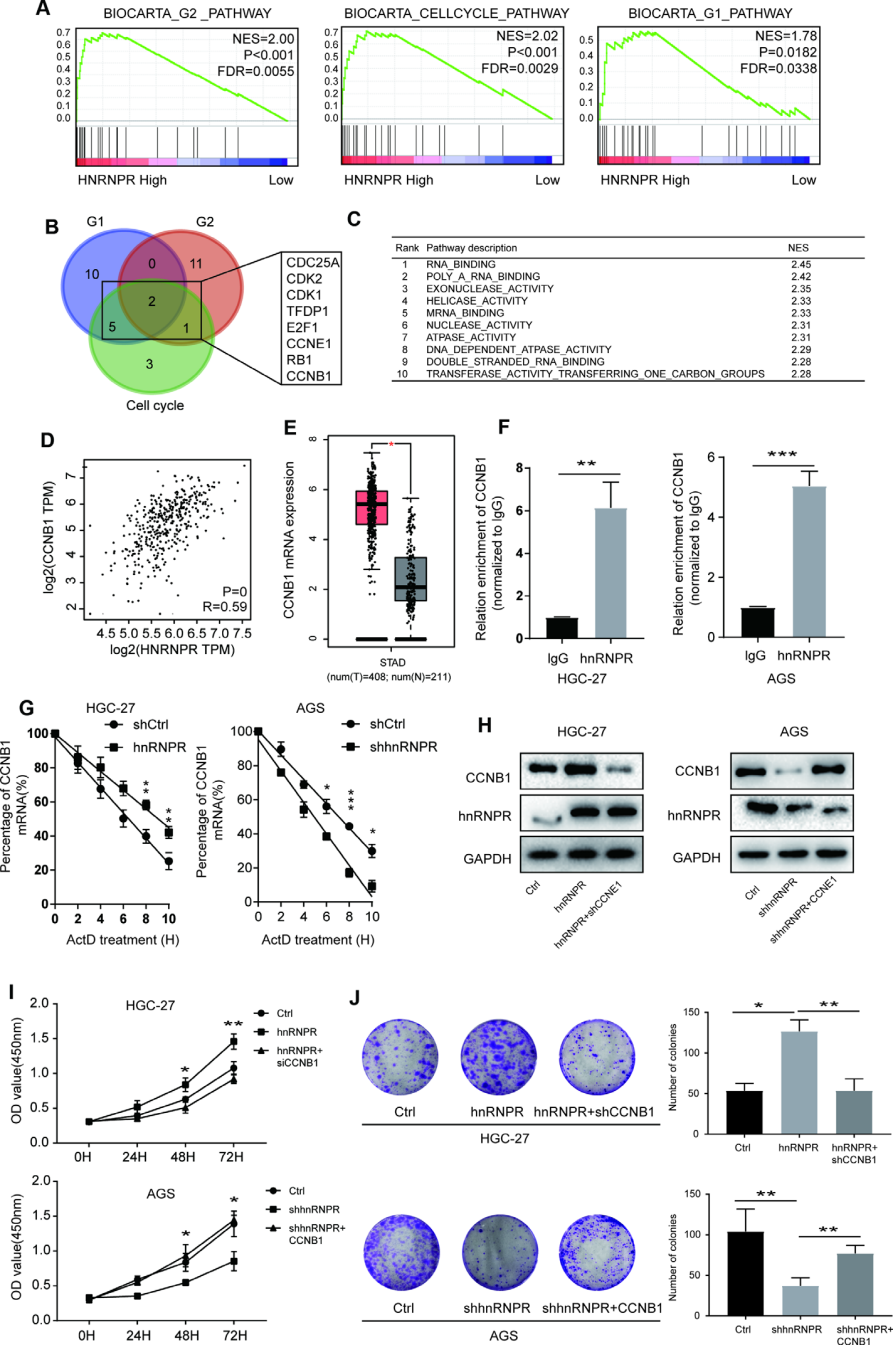
**hnRNPR promoted gastric cell proliferation by binding CCNB1 mRNA.** (**A**) GSEA showed that high hnRNPR expression was positively correlated with cell cycle pathway, G2 pathway, and G1 pathway. (**B**) Venn graph revealed that CDC25A, CDK2, CDK1 TFDP1, E2F1, CCNE1, RB1, CCNB1 overlapped in the three groups. (**C**) Pathway enrichment analysis showed that “RNA binding” is the top one with significance. (**D**) The expression of hnRNPR was positively correlated with the expression of CCNB1. (**E**) The level of CCNB1 was higher in tumors than that in normal tissues. (**F**) RIP-PCR indicated that CCNB1 mRNA is significantly increased hnRNPR groups in HGC-27 and AGS cell lines. (**G**) CCNB1 mRNA stability analysis in HGC-27 and AGS cells after actinomycin D (ActD) treatment. Cells were transfected with hnRNPR or shhnRNPR or a control. Cells were harvested at the indicated timepoints. Expression levels were normalized to “0 h” and GAPDH was used as reference gene. (**H**) HGC-27-control and HGC-27-hnRNPR cells were transfected with shRNA control or shRNA against CCNB1 for western blot, and AGS-control and AGS-shRNPR cells transfected with control or CCNB1 plasmid for western blot. (**I**) CCK8 and (**J**) colony formation assays showed that CCNB1 knockdown partially attenuated the enhanced cell proliferation induced by hnRNPR overexpression in HGC-27 cells, while CCNB1 overexpression partially rescued the inhibition of cell growth induced by hnRNPR silencing in AGS cells. Data are from three independent experiments performed in triplicate. P values were calculated with two-tailed unpaired Student’s t test. The expression correlation was determined with Pearson’s correlation analysis. *, P<0.05, **, P<0.01.

To further explore the molecular mechanisms underlying hnRNPR-mediated phenotype, GSEA enrichment analysis based on the TCGA STAD was performed to identify relevant pathways. G1, G2, and cell cycle signatures were identified as the significant pathways affected by hnRNPR (Figure 3A, Supplementary Table 3, Supplementary Figure 3A, 3B), indicating that hnRNPR regulates cell cycle. Bioinformatic analysis showed that 17 genes, 13 genes, and 11 genes were regulated by hnRNPR in G1, G2, and cell cycle pathways, respectively (Supplementary Table 3). To identify the molecules in these pathways that may be regulated by hnRNPR, a Venn diagram was constructed and revealed that eight of these genes (CDC25A, CDK2, CDK1 TFDP1, E2F1, CCNE1, RB1, CCNB1) were regulated in at least two pathways (Figure 3B). Because hnRNPR is an RNA binding protein defined by the Gene Ontology terms “RNA binding” and “mRNA binding” in the Kyoto Encyclopedia of Genes and Genomes (KEGG) [23]. GESA analysis also demonstrated a GO term “RNA binding” as the top term (Figure 3C). As shown in Figure 3D and Supplementary Figure 3A, the expression of the eight genes was positively correlated with the expression of hnRNPR and the levels of CDC25A, CDK1, TFDP1, E2F1, CCNE1, RB1, CCNB1 were remarkably higher in the tumor than in normal controls, except for CDK2 (Figure 3E, Supplementary Figure 3C, 3D). To confirm that high expression of CCNB1 was associated with direct binding of hnRNPR to CCNB1 mRNA, RNA immunoprecipitation (RIP)-PCR assay was performed to examine the expression of candidate genes. After analysis of the isolated copurifying RNA revealed that CCNB1 mRNA was detected with significance and the rest of candidate genes without significance in the hnRNPR group in two cell lines (Figure 3F, Supplementary Figure 3E). Further experiments were performed to determine whether hnRNPR affected the stability of genes in GC cells. Subsequent to shRNA-directed hnRNPR inhibition, AGS cells were treated with Actinomycin D and then subjected to qRT-PCR. The results showed that only CCNB1 expression was reduced. With the treatment of Actinomycin D, the mRNA level of CCNB1 continuously enhanced in the HGC-27-hnRNPR cells compared to that in the control cells (Figure 3G). The results validated the specific binding of hnRNPR with CCNB1 mRNA in GC cells. Hence, hnRNPR directly associated with CCNB1 mRNA, stabilized this transcript, and enhanced CCNB1 expression. The expression of CCNB1 decreased at mRNA and protein levels in hnRNPR-knocked-down cells. The expression of CCNB1 was decreased in hnRNPR-overexpression cells by siRNA and the expressed CCNB1 in hnRNPR-knockdown cells was forced (Figure 3H). Silencing CCNB1 significantly reduced the cell proliferation and clone formation ability of hnRNPR-overexpression cells, whereas overexpression of CCNB1 markedly attenuated the clonogenicity in hnRNPR-knockdown cells (Figure 3I, 3J). The results of flow cytometry also showed that hnRNPR promoted G2/M transition phase of the cell cycle by upregulation CCNB1 (Supplementary Figure 3F), These findings indicated that hnRNPR enhanced the cell proliferation of gastric cancer cells via maintaining CCNB1 stability.

### HnRNPR enhances the stability of CENPF and promotes tumor metastasis

**Figure 4:**
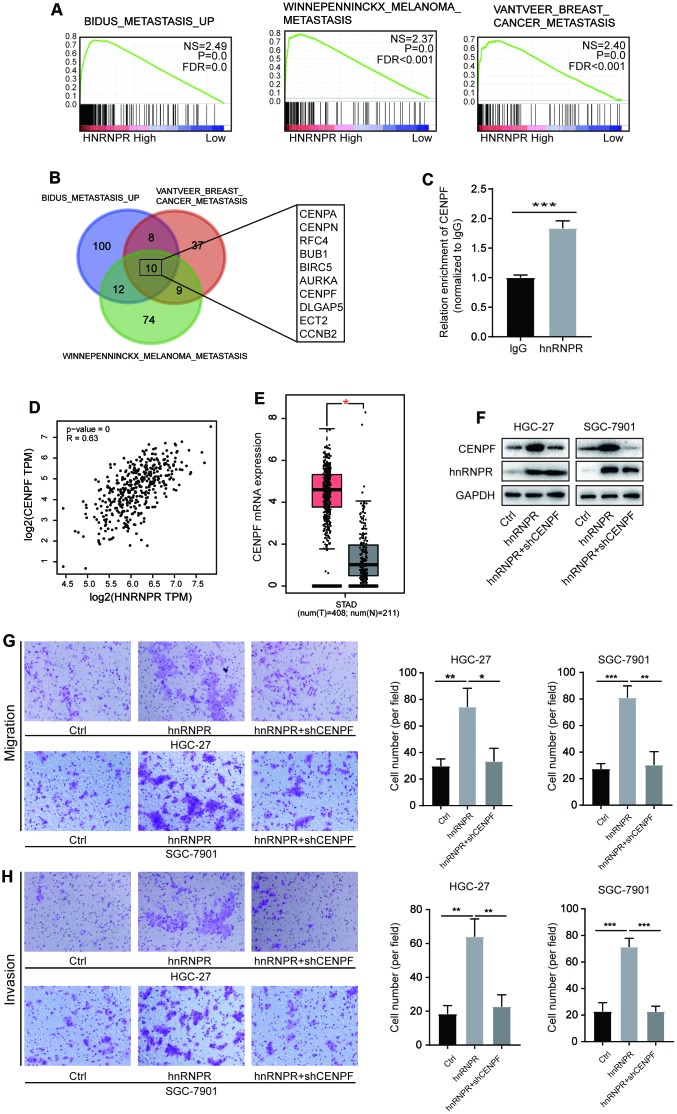
**HnRNPR promoted cancer aggressiveness by binding CENPF mRNA.** (**A**) GSEA analysis indicated that high hnRNPR expression positively correlated with three metastasis signatures. (**B**) Venn graph indicated that CENPA, CENPN, RFC4, BUB1, BIRC5, AURKA, CENPF, DLGAP5, ECT2, and CCNB2 were overlapped in the three groups. (**C**) The expression of CENPF was positively correlated with that of hnRNPR. (**D**) The CENPF expression in gastric tumors was significantly higher than that in normal tissues in TAGC-STAD database. (**E**) RIP-PCR revealed that CENPF RNA is enriched in the hnRNPR group compared to the control. (**F**) Two (HGC-27 and SGC-7901) cell lines control and hnRNPR cells were transfected with shRNA control or shRNA against CENPF for Immuno-blotting. (**G**) Migration and (**H**) invasion assays showed that inhibition of CENPF rescued the aggressiveness induced by hnRNPR in SGC7901 and HGC-27 cell lines. Data were from three independent experiments performed in triplicate. P values were calculated with two-tailed unpaired Student’s t test. The expression correlation was determined with Pearson’s correlation analysis. *, P<0.05, **, P<0.01.

Although CCNB1 has been found to be a critical downstream target of hnRNPR, and plays a role in the cell cycle, few studies focused on the role of CCNB1 in the tumor metastasis, thus we performed Transwell with/without Matrigel experiments to detect the function of CCNB1 on metastasis of gastric tumor cells. We found that CCNB1 overexpression or inhibition has no effect on tumor migration and invasion (Supplementary Figure 4A), implying the presence of other unknown targets in gastric cancer cells. Thus, we attempted to establish whether hnRNPR interacts with other targets in cancer metastasis. GSEA analysis revealed that hnRNPR was remarkably enriched in metastatic processes (Figure 4A). Venn diagram showed that ten of these genes (CENPA, CENPN, RFC4, BUB1, BIRC5, AURKA, CENPF, DLGAP5, ECT2, CCNB2) were involved in three pathways (Figure 4B, Supplementary Table 4). RIP-PCR analysis showed that CENPF mRNA, not other genes directly bound to the hnRNPR protein (Figure 4C, Supplementary Figure 4B). Similarly, TCGA STAD dataset implied that the expression of hnRNPR was positively correlated with the level of CENPF (Figure 4D). Furthermore, CENPF expression was significantly upregulated in the tumor compared with the correspondent controls (Figure 4E). The level of CENPA, CENPN, RFC4, BUB1, BIRC5, AURKA, DLGAP5 and ECT2 were correlated to hnRNPR and increased in the tumor tissues (Supplementary Figure 4B, 4C), suggesting that hnRNPR indirectly regulated these genes to promote metastasis. To verify whether CENPF could, at least in part, rescue the cell invasion phenotype due to hnRNPR downregulation of, CENPF was downregulated in hnRNPR-overexpressing cells (Figure 4F). Consistent with our previous findings, cell migration and invasion ability were enhanced by up-regulation of hnRNPR expression, but substantially impaired after inhibition of CENPF protein (Figure 4G, 4H). In summary, these results demonstrated that hnRNPR enhanced cell aggressiveness by positively regulating CENPF.

### Deregulation of hnRNPR suppresses the tumorigenicity and metastasis in vivo

**Figure 5:**
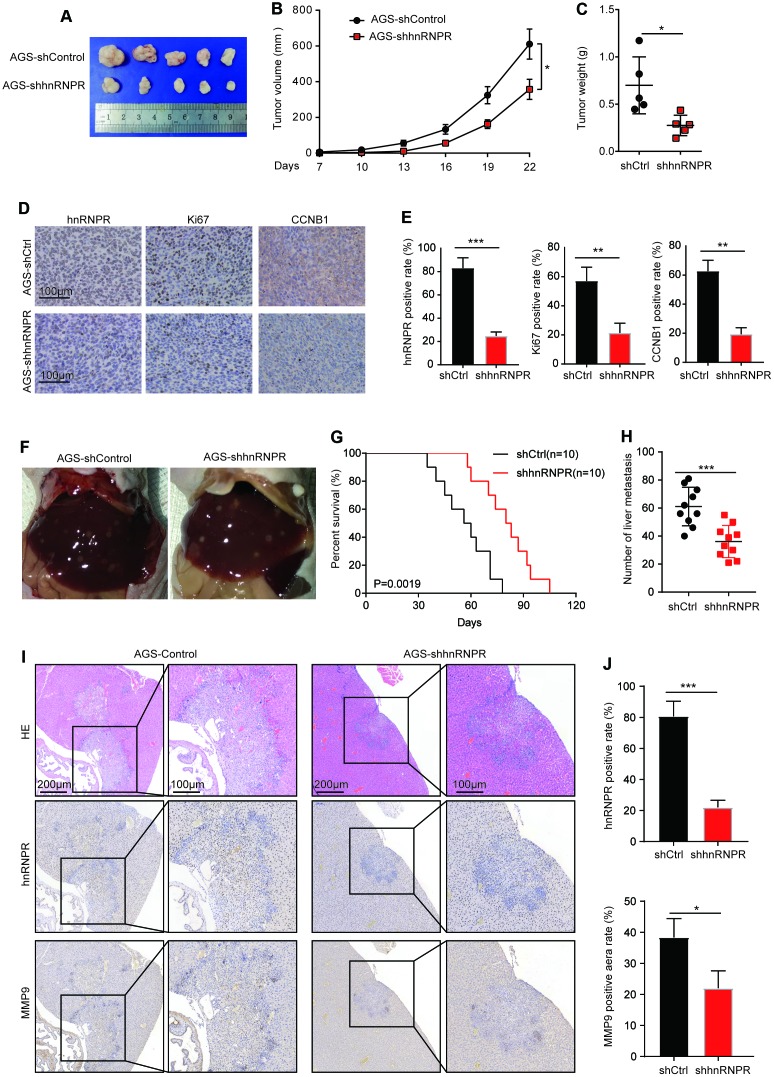
**Repression of hnRNPR inhibits tumor growth and metastasis in vivo.** (**A**) Tumor size and (**B**) tumor volumes in hnRNPR-knockdown and control groups. (**C**) Tumor weight in hnRNPR-knockdown and control groups (n=5). (**D**) Representative images (**E**) quantification of hnRNPR, Ki67, and CCNB1 in the indicated xenograft tumors. (**F**) Representative images of liver metastasis in the indicated tumors. (**G**) Kaplan–Meier curve of mice showing low expression of hnRNPR versus high expression of hnRNPR group. (**H**) Number of liver metastasis (**I**) representative image (**J**) Quantification of hnRNPR and MMP9 expression in the indicated xenograft tumors. P values were calculated with two-tailed unpaired Student’s t-test, or log rank Mantel-Cox test. *, P<0.05, **, P<0.01.

To evaluate the effect of hnRNPR on cell tumorigenicity in vivo, AGS cells with hnRNPR- knockdown (AGS-shhnRNPR) were implanted into nude mice, and cells transfected with empty lentiviral vectors served as the control. The volumes of tumors formed by the AGS-shhnRNPR cells were remarkably smaller than those formed by control cells (Figure 5A). After 22 days, the size and weight of the xenograft tumors were assessed after euthanasia. As expected, tumors formed by AGS-shhnRNPR cells were markedly smaller and lighter than tumors formed by control cells (Figure 5B, 5C). According to IHC staining results, the Ki67-positive rates in xenograft tumors formed by the AGS-shhnRNPR cells that significantly lower than those formed by controls (Figure 5D). Furthermore, it was that the expression of CCNB1 was downregulated in AGS-shhnRNPR groups. Quantitative analysis revealed that the staining score in control tumor was remarkably higher than that of shhnRNPR cells (Figure 5E, 5J). These results indicated that hnRNPR inhibition impairs GC growth in vivo.

To further confirm the role of hnRNPR-mediated metastasis in vivo, the spleens of ten mice in each group were injected with AGS-shhnRNPR cells and AGS-shControl cells. The KM plot revealed that low expression of hnRNPR prolonged the mice survival in GC models (Figure 5F, 5G). The number of metastatic nodules in the liver surfaces were counted. A remarkably smaller number of metastatic nodules were generated at the surface of the liver of mice injected with AGS-shhnRNPR cells (Figure 5H). H&E staining confirmed that the nodules in the liver were metastatic tumors in all mice (Figure 5I). IHC staining was carried out to validate that the level of hnRNPR originated from the shhnRNPR cells developed remarkably slower than those from controls in the liver (Figure 5I). The hnRNPR-positive cells or MMP9 positive areas in shhnRNPR tumor were remarkably reduced than those in the control tumors (Figure 5I). Taken together, these results showed that hnRNPR controlled tumorigenicity and metastasis in vivo. This showed that hnRNPR promoted the aggressiveness of tumors, which was consistent with the observation in vitro experiments.

### Clinical association of hnRNPR expression with the level of CCNB1 and CENPF in human GC samples

**Figure 6:**
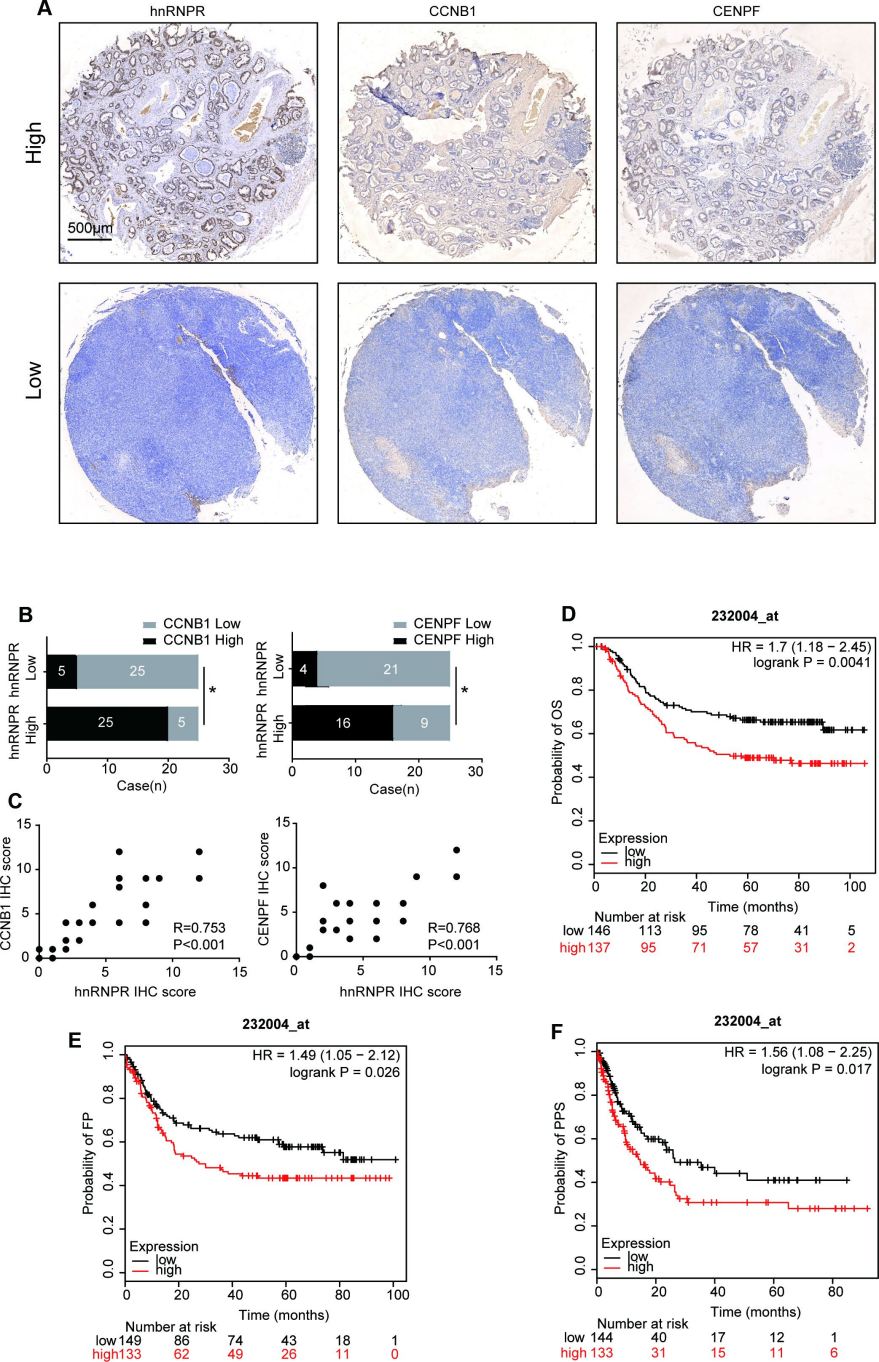
**hnRNPR expression in the clinical samples.** (**A**) Two representative images showing low or high expression of hnRNPR, CCNB1, and CENPF in human GC tissues. Scale bars 500μm. (**B**) Human gastric cancer tissues were used for hnRNPR and CCNB1/CENPF staining by IHC and quantitated. Chi-square test was used to analyze the correlation between HnRNPR and CCNB1/CENPF. P<0.05 was considered as significant. (**C**) Association between IHC score of hnRNPR and CCNB1/CENPF expression. (**D**–**F**) Kaplan Meier curves of OS (overall survival), FP (first progression), and PPS (post-progression survival) in a cohort of gastric cancer patients stratified by hnRNPR expression.

The relationship between the protein expression of hnRNPR and the level of CCNB1 and CENPF was tested in a cohort of 50 human GC samples by immunohistochemistry. Representative images of CCNB1 and CENPF were shown in Figure 6A. GC patients with high levels of hnRNPR in the tumors exhibited high expression of CCNB1 and CENPF (Figure 6A, 6B). Moreover, Pearson’s correlation analysis not only suggested a positive correlation between the protein level of CCNB1 and hnRNPR (R=0.753, P<0.001), but also a positive correlation between the protein level of CENPF and hnRNPR (R=0.768, P<0.001) (Figure 6C).

To assess whether hnRNPR could be related to the prognosis of GC patients, we found that hnRNPR mRNA high expression was found to be correlated with poor overall survival for GC patients (Affymetrix ID: 232004_at, HR=1.7(1.18-2.45), logrank P=0.0041) in the GSE62254 dataset [24]. Similarly, the time of first progression (FP) in the hnRNPR^high^ group is significantly earlier than that in the hnRNPR^low^ group (Affymetrix ID: 232004_at, HR=1.49(1.05-2.12), logrank P=0.026). Furthermore, the probability of post-progression survival (PPS) in the hnRNPR^high^ groups is remarkably decreased than that in the hnRNPR^low^ group (Affymetrix ID: 232004_at, HR=1.56(1.08-2.25), logrank P=0.017) (Figure 6D–6F). Overall, clinical datasets analysis confirmed that hnRNPR level positively correlated with CCNB1 and CENPF, and high expression of hnRNPR is associated with a bad prognosis. Thus, these findings confirmed that the hnRNPR-CCNB1/CENPF axis increased metastatic potential of GC cells.

## DISCUSSION

**Figure 7:**
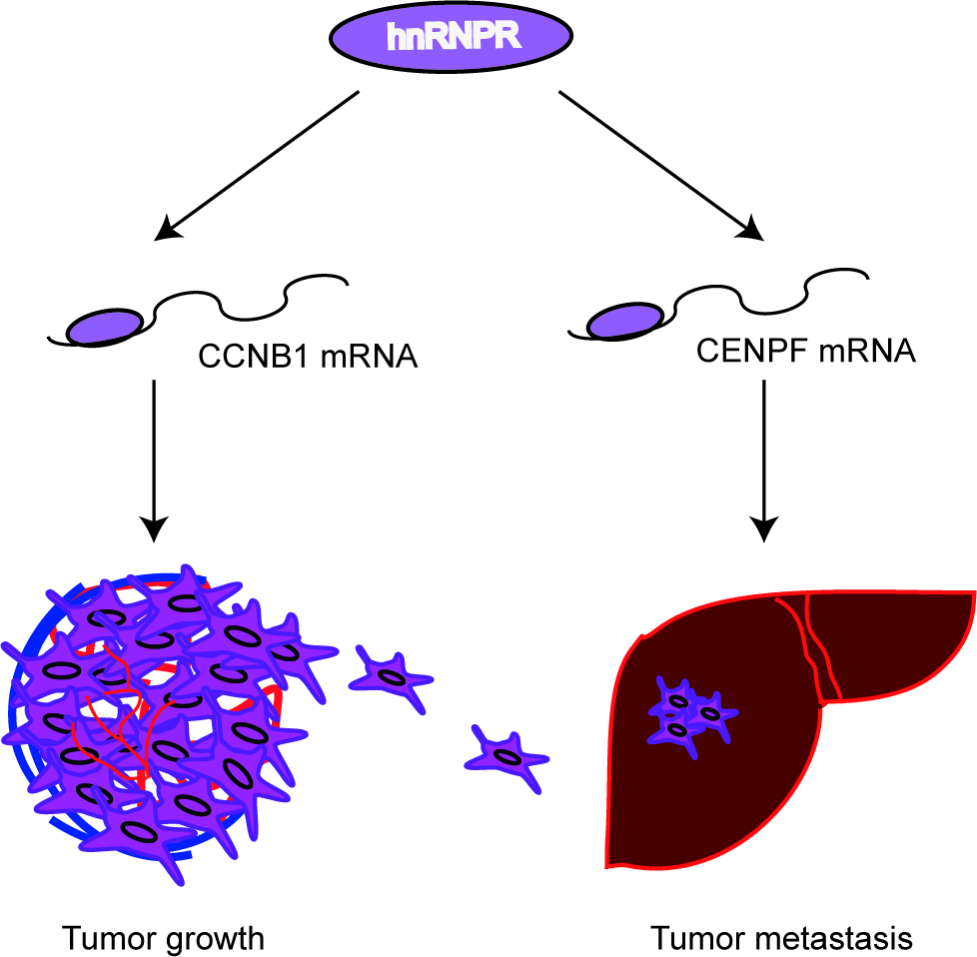
**Proposed model: hnRNPR protein directly binds to CCNB1/CENPF mRNA to enhance its stability, leading to increased cell proliferation and invasiveness in gastric cancer.**

To the best of our knowledge, although many studies have shown that hnRNPs play an important role in tumor progression, the pro-oncogene role of hnRNPR in cancer progression has not been reported. In this study, we found that hnRNPR was highly expressed in GC specimens than that in peritumoral control samples by bioinformatic analysis. Based on in vitro and in vivo models, it was found that hnRNPR increased cell proliferation, invasion, and migration. Furthermore, hnRNPR protein directly interacts with CCNB1 and CENPF mRNA. Therefore, these results indicated that hnRNPR acts as a pro-oncogene in GC development (Figure 7).

It was important to investigate the molecular mechanisms of hnRNPR overexpression in GC. A series of gain-and loss-function experiments in GC cell lines revealed that hnRNPR dramatically accelerated cell cycle progression. To elucidate the mechanism of hnRNPR in the cell cycle data obtained from TCGA stomach adenocarcinoma was analyzed by GESA tools. We identified G1, G2 and cell cycle pathways were significant critical downstream of hnRNPR. It is known that cell cycle deregulation is a common hallmark of human cancer [25, 26]. Several therapeutic strategies have been targeting cell division cycle in cancer [27]. In this study, hnRNPR level was positively associated with the expression of eight genes (CDC25A, CDK2, CDK1 TFDP1, E2F1, CCNE1, RB1, CCNB1) in TCGA GC cohort. In addition, the direct binding between hnRNPR protein and CCNB1 mRNA was confirmed by RIP-PCR analysis. It is well known that CCNB1 is a mitotic-specific factor and forms complexes with CDK1 to regulate G2-M transition. Recently, accumulating evidence has indicated that the expression of CCNB1 may be correlated with aggressive tumor ability and poor outcome in cancer patients [28–30], including GC [31], which is consistent with the findings in this study. Ectopic hnRNPR expression increased CCNB1 expression, while knockdown of hnRNPR decreased CCNB1 expression. Rescue experiments demonstrated that pro-proliferation effect of hnRNPR required CCNB1, since silencing of CCNB1 partially reversed the effect of hnRNPR in GC cells.

The results also showed that hnRNPR overexpression increased cell migration and invasion, whereas hnRNPR inhibition decreased cell motility and invasiveness. Bioinformatics correlation analysis showed a significant correlation between hnRNPR level and the expression of tumor metastasis-related genes in TCGA GC cohort, such as CENPA, CENPN, RFC4, BUB1, BIRC5, AURKA, CENPF, DLGAP5, ECT2, and CCNB2. RIP-PCR analysis showed that hnRNPR interacted with CENPF and increased its mRNA stability. These results demonstrated that hnRNPR increased the protein level of CENPF by increasing its RNA stability. CENPF is a member of the kinetochore family, which regulates tumoral proliferation in various cancers. CENPF is frequently overexpressed in pancreatic cancer [32], hepatocellular carcinoma [33], prostate cancer [34], and metastatic prostate cancer [35, 36]. This study suggested hnRNPR overexpression enhanced the level of CENPF and CENPF inhibition abrogated the effect of hnRNPR on the invasive ability of GG cells.

Using the subcutaneous in vivo model, it was observed that hnRNPR markedly slowed the tumor proliferation rate and impaired tumor growth, this was consistent with the finding that hnRNPR acted as an oncogene in vitro. Based on liver metastasis model created by intra-spleen injection, it was found that hnRNPR knockdown decreased the number of metastatic nodules and tumor size. The expression of hnRNPR, CCNB1 and CENPF were detected in the clinical specimens. This work uncovered a novel relationship between hnRNPR and CCNB1/CENPF in the GC samples. The patients with high hnRNPR expression tended to have a high expression of CCNB1/CENPF.

In summary, the current results demonstrate that hnRNPR regulates GC development via binding to CCNB1 and CENPF mRNA. High expression of hnRNPR in GC strongly correlates with tumor aggressiveness. In conclusion, these findings suggest that hnRNPR-CCNB1/CENPF axis may be a potential therapeutic target for the management of GC patients.

## MATERIALS AND METHODS

### Clinical patients tissue microarray analysis

A total of 50 formalin-fixed paraffin-embedded (FFPE) gastric cancer tissues were obtained from Zhongshan Hospital, Fudan University. The slides were blocked and then incubated overnight at 4°C with primary antibody (listed in Supplementary Table 1). Tissue sections were then incubated with biotinylated goat anti-rabbit or anti-mice immunoglobulin at 37°C for 40 min. Finally, the sections were stained with diaminobenzidine (DAB). Each section was evaluated by stained sections in three representative areas and then analyzed to determine the stained proportion and density of positive tumor cells by three independent pathologists who were blinded to the clinical variances and prognosis of the patients. For each section, the proportion of hnRNPR-positive cells was scored from 0% to 100% (based on the extent of positive staining in each microscopic field of view: (0=0%; 1 =1-5%; 2, 6-29%; 3, 30-59%; 4, 59%-100%). The staining intensity varied from negative to strong: negative (0), low (1), medium (2), and high (3). The final score was calculated by multiplying these two scores**.** The expression level of hnRNPR was considered as high at a final score >6, and as low expression at the final score < 6. The study was approved by the Research Ethics Committee of Zhongshan Hospital.

### Cell culture and animal

Five gastric cancer cell lines SGC-7901, AGS, HGC-27, MKN-28, MGC-803 and one immortalized normal gastric mucosal epithelial cell line (GES-1) used in this study were purchased from Cell Bank of the Chinese Academy of Sciences (Shanghai, China). All cells were cultured in RPMI 1640 medium containing 10% FBS (Gibco, USA) in a humidified atmosphere with 5% CO_2_ at 37°C. 4–6 weeks-old male BALB/c nu/nu mice (Shanghai Institute of Material Medicine, Chinese Academy of Science) were maintained under specific-pathogen-free conditions.

### In vivo tumorigenesis model

Subcutaneous xenograft tumor model was used for in vivo tumor growth assays. 5×10^6^ AGS-shcontrol (AGS-shCtrl), and AGS-shRNA-hnRNPR (AGS-shhnRNPR) cells were injected subcutaneously into the armpit of nude mice. The subcutaneous tumor volumes were calculated with digital calipers, every three days after one week. The volumes of tumors (V, cm^3^) were measured as follow; V=Width^2^ x Length/2. Twenty-two days later, all the mice were sacrificed and tumor tissues were finally weighed. Intra-splenic injection model was used for in vivo metastasis experiment. Laparotomy was performed after anesthesia, and the 1×10^6^ AGS-shCtrl or AGS-shhnRNPR cells were slowly injected into the spleen. After 2 minutes, a splenectomy was performed followed by a closure of abdominal incision. The overall survival was observed up to 105 days until all mice died. Tumor nodules formed in the liver were counted under a dissecting microscope. Animal protocols were approved by the Institutional Animal Care and Use Committee of the Zhongshan Hospital.

### mRNA stability assay

The cells transfected with shhnRNPR, hnRNPR or Ctrl were added into 6-well plates. After 24h, actinomycin D (ActD; 20ug/mL) was added to the culture medium to inhibit mRNA transcription activity, and cells were harvested at the indicated time points (2, 4, 6, 8, 10h) for total RNA extraction. The total RNA was subjected to qRT-PCR to analyze mRNA stability.

### RNA immunoprecipitation

EZ Magna RNA immunoprecipitation Kit (Millipore, USA) was applied according to the manufacturer’s protocol. Briefly, AGS cells were lysed in immunoprecipitation lysis buffer. Magnetic beads were conjugated with anti-human argonaute 2 (Ago2) antibody or control anti-lgG at room temperature for 30min, and the cell extract were incubated with magnetic beads for 6h at 4°C. The RNA quality was assessed using a bioanalyzer. The immunoprecipitated RNA was extracted and performed by qRT-PCR to investigate the expression of candidate genes.

### Immunoblot analysis

Equal amounts of proteins were extracted from the whole cell lysates, resolved by 10% SDS-PAGE and electrophoretically transferred to PVDF membranes (Millipore, USA). The membranes were blocked in 5% non-fat milk for 2 h and then incubated with the primary antibodies. Next, the membranes were incubated with peroxidase-conjugated second antibody. Antibody detection was performed by addition of enhanced chemiluminescence. The primary antibodies were shown in the Supplementary Table 2. GAPDH served as the endogenous reference.

### Quantitative RT-PCR (qRT-PCR)

The cells were collected, and total RNA was extracted using TRIzol (Invitrogen, USA). And then reverse-transcribed to cDNA by the PrimeScript RT reagent Kit (Takara, Japan). the expression of mRNA was detected with SYBR Green Real-time PCR Master Mix (Takara, Japan). The relative expression of candidate gene was measured as the fold change using 2^−ΔCT^ formula. The expression of GAPDH was used as an endogenous reference for normalization. The primers used are provided in Supplementary Table 1.

### Lentiviruses construction, plasmid and cell transfection

Lentiviral containing shRNA targeting hnRNPR and a negative lentiviral vector were purchased from Shanghai Genechem (Shanghai, China). HnRNPR cDNA was cloned into the pcDNA3.1 vector to construct an expression vector and an overexpressing-lentiviruses. The viruses were transfected into GC cells as per the recommendations of the manufacturer. CCNB1 and CENPF siRNAs were purchased from Invitrogen. GC cells were transfected with 50nM siRNA using Lipofectamine 3000 (Invitrogen, USA) according to the manufacturer’s protocols.

### Wound scratch analysis

Briefly, gastric cells at 90% confluence were cultured in 12-well plates with complete medium. The cells were scratched after 12 h of incubation to create a wound and then washed three times with PBS. The wound recovery widths at 0 h (W1) and at 24 h (W2) were observed and recorded under a microscope. The relative cell relative migration rate was calculated using the following formula: (W2-W1)/W1 x 100%.

### Migration and invasion assays

The metastatic ability of the cells was investigated by Transwell plates (Corning, USA). Serum-free single-cell suspensions were seeded on the upper chambers per well. About 500 uL of 1640 medium containing 10% FBS was used as a chemoattractant in the lower chamber. For invasion assays, the membrane inserts were pre-coated with Matrigel. The cells were cultured for 24h and stained with 0.5% crystal violet. Finally, the cells that migrated or invaded across the membrane were counted.

### Cell proliferation assay

GC cells vitality was investigated by CCK-8 assay kit (Dojindo, Japan). The cells in 100 uL culture medium were added into 96-well plates (2 x10^3^ cells/well) and incubated with 100 for 4 days. An equal number of cells were added into 6-well plates and incubated with a complete medium. After 1 week of cell culture, the clones formed were fixed, stained and calculated.

### Gene set enrichment analysis (GSEA) and bioinformatics analysis

To investigate the cancer-related molecular pathways affected with hnRNPR in gastric cancer, the GSEA was applied based on TCGA-STAD. GSEA v3.0 tool was used to assess the relationship between particular gene sets from the MSigDB database and hnRNPR [37, 38]. The number of gene set permutations were 1000 times for each analysis, and the threshold for the nominal P value and FDR value were set to 0.05 for normalized enrichment score (NES). The informatic analysis of the gene profiles and relevant genes correlation were performed using the online website (GEPIA) (http://gepia.cancer-pku.cn/index.html) [39].

### Statistical analysis

SPSS 23.0 (SPSS, USA) software and GraphPad Prism 7 (GraphPad, USA) were used for statistical analysis. All data were presented as mean + SD. The differences in hnRNPR expression between two groups were determined by Student’s t-tests (two-tailed). The correlations between hnRNPR and other gene expression were determined by Pearson’s rank correlation analysis.

## Supplementary Material

Supplementary Figures

Supplementary Tables 1-3

Supplementary Table 4
