# Supplementary material for: HnRNPR-CCNB1/CENPF axis contributes to gastric cancer proliferation and metastasis
Source: Aging (Albany NY). 2019 Sep 16;11(18):7473–91. doi: 10.18632/aging.102254 (PMC6782008; doi:10.18632/aging.102254)
Supplement: Supplementary Figures [file aging-11-102254-s003.pdf]

SUPPLEMENTARY FIGURES

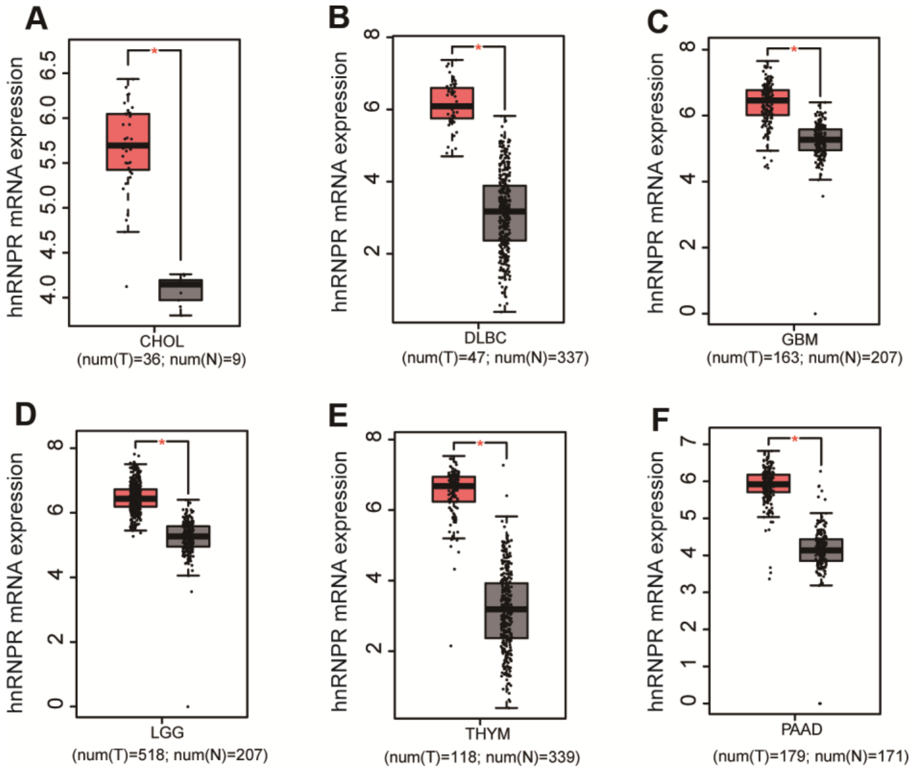

Supplementary Figure 1. hnRNPR is upregulated in many cancers. The GEPIA database revealed that hnRNPR mRNA expression was overexpressed in CHOL (**A**) DLBC (**B**) GBM (**C**) LGG (**D**) THYM (**E**) PAAD (**F**). Abbreviation: GEPIA: gene expression profiling interactive analysis; CHOL: Cholangio carcinoma; DLBC: Lymphoid Neoplasm Diffuse Large B-cell Lymphoma; GBM: Glioblastoma multiforme; LGG: Brain Lower Grade Glioma; THYM: Thymoma; PAAD: Pancreatic adenocarcinoma.

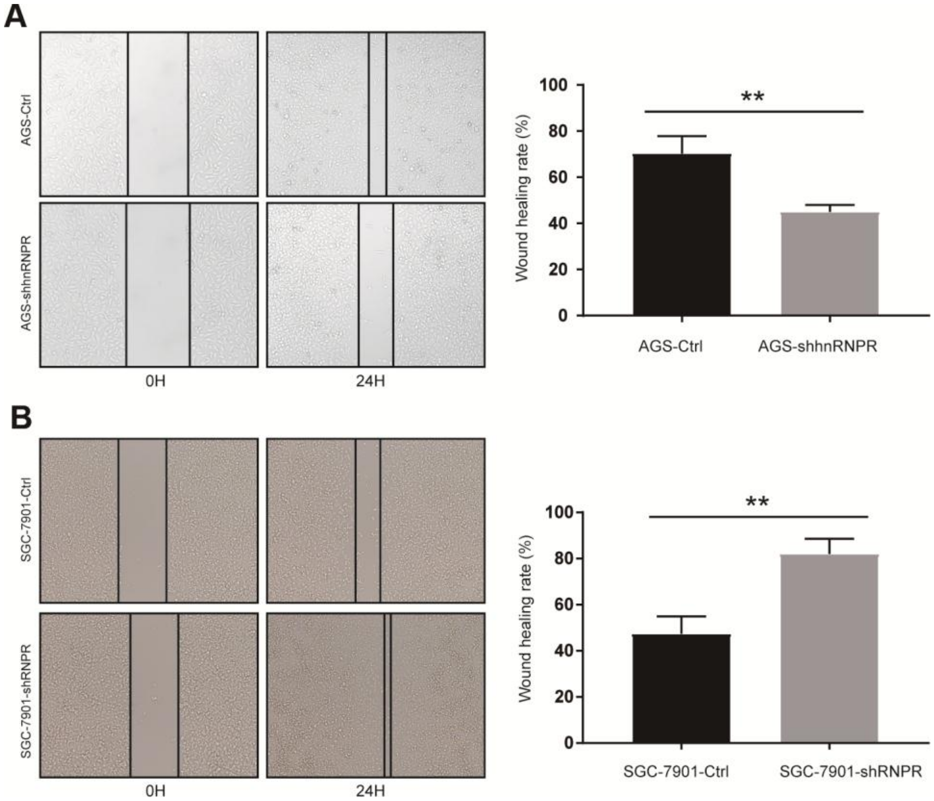

Supplementary Figure 2. hnRNPR promoted cell migration. (**A**) Knock-down hnRNPR expression in AGS cell compromised their migration ability. (**B**) Overexpression of hnRNPR in SGC-7901 cells increased their migration ability. Each experiment was performed in triplicate and repeated for three times. P values were calculated with two-tailed unpaired Student's t-test. **, P<0.01.

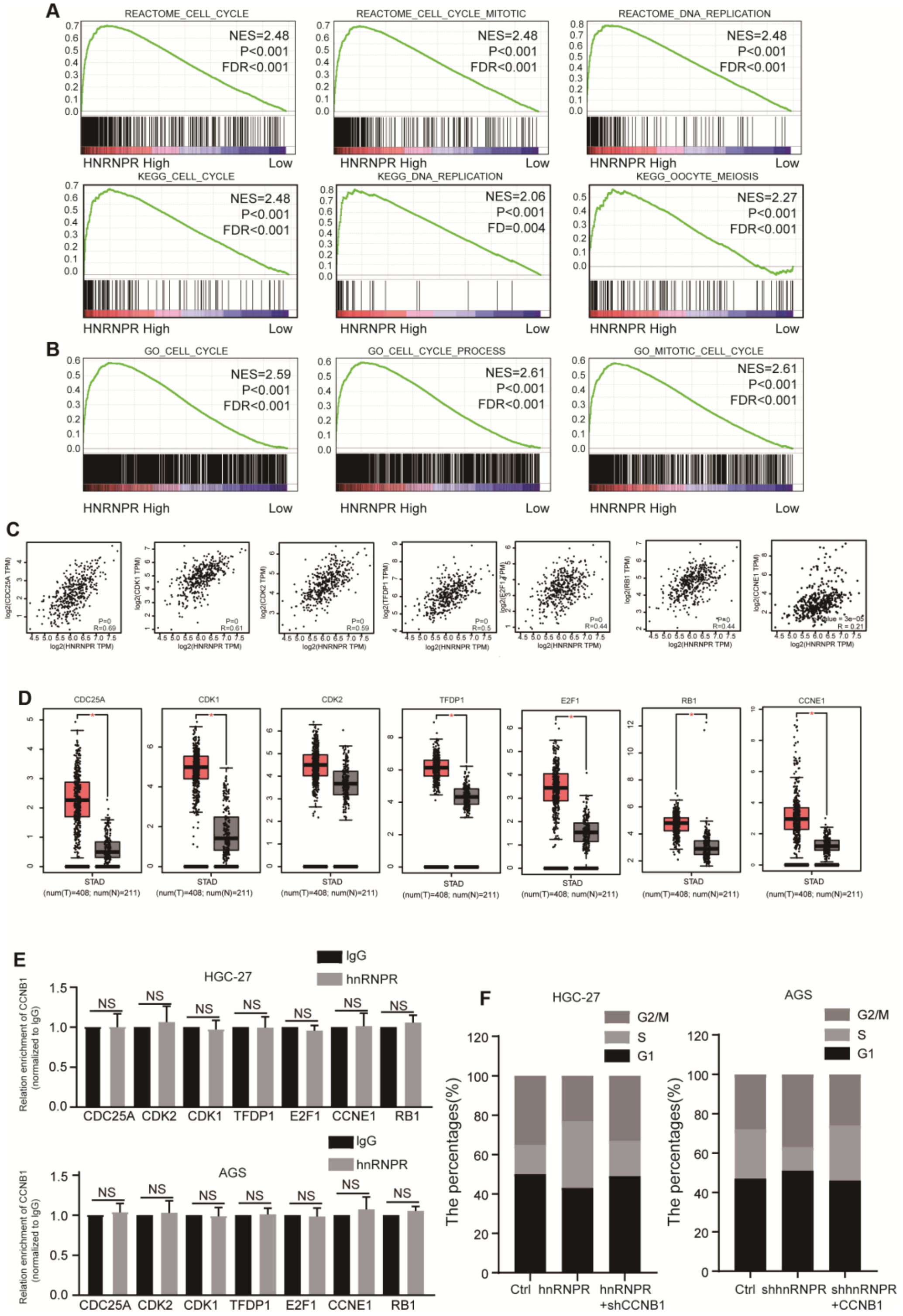

Supplementary Figure 3. hnRNPR is positively correlated with the expression of several cell cycle regulator factors. (**A**) The hnRNPR expression is correlated with cell cycle in REACTOME and KEGG database. (**B**) GO analysis indicated that the hnRNPR is involved in the cell cycle. (**C**) GEPIA revealed that the level of CDC25A, CDK2, CDK1 TFDP1, E2F1, RB1, CCNB1 was positive correlated with the expression of hnRNPR. (**D**) GEPIA indicated that the expression of CDC25A, CDK1 TFDP1, E2F1, RB1, CCNB1 were upregulated in TCGA STAD, whereas the level of CDK2 is increased without significance. The expression correlation was determined with Pearson's correlation analysis. *, P<0.05. (**E**) RIP-PCR indicated that CDC25A, CDK2, CDK1, TFDP1, E2F1, CCNE1 and RB1 mRNA were bound to hnRNPR in HGC-27 and AGS cell lines. (**F**) overexpression of CCNB1 decreased the percentage of cells in the G2/M phase and CCNB1 knockdown induced the G2/M arrest. NS. No significance.

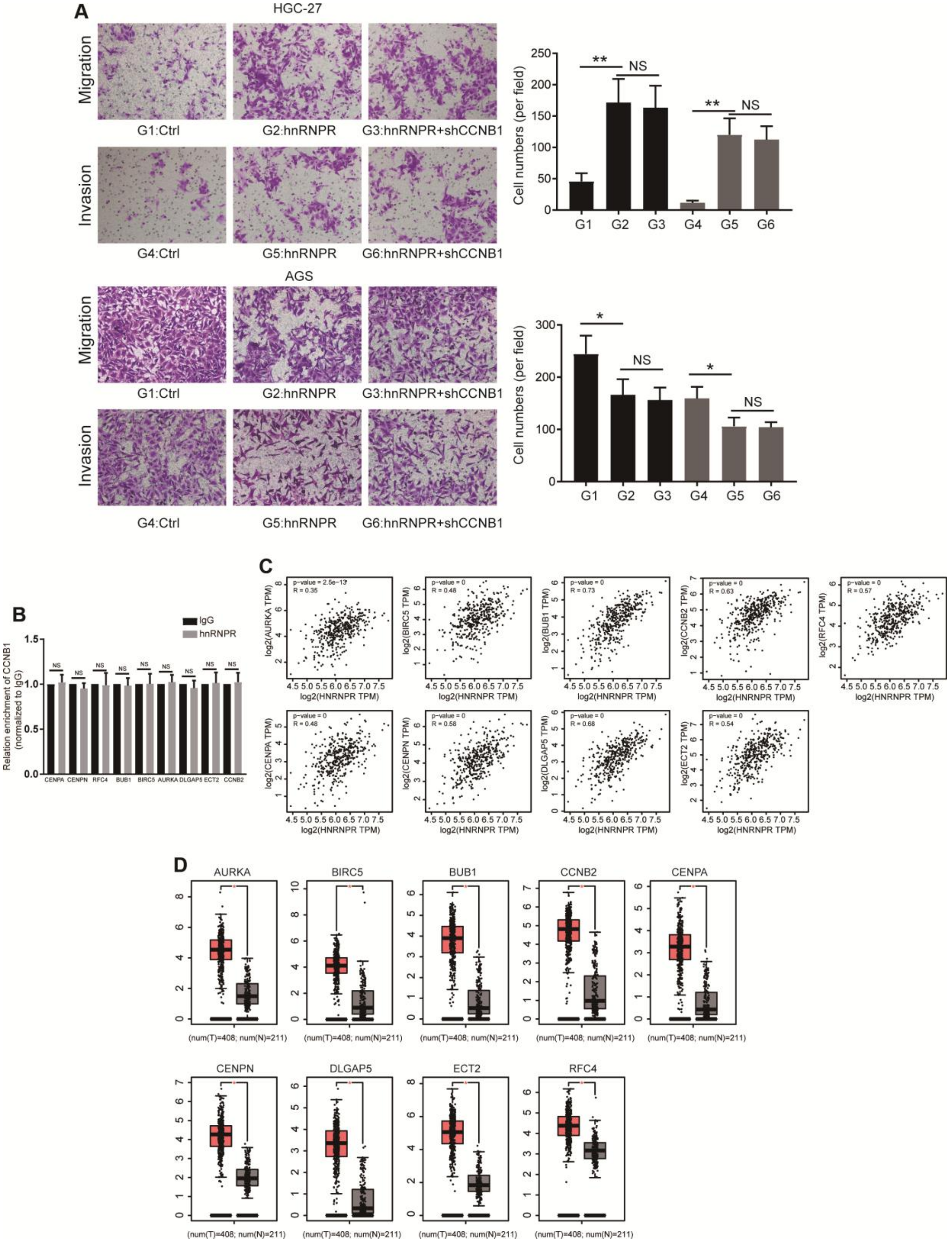

Supplementary Figure 4. hnRNPR positively associates with the expression of some molecules that regulate tumor metastasis. (**A**) CCNB1 had no effect on tumor invasion and metastasis induced by hnRNPR. (**B**) RIP-PCR indicated that CENPA, CENPN, RFC4, BUB1, BIRC5, AURKA, DLGAP5, ECT2, and CCNB2 mRNA were bound to hnRNPR. (**C**) GEPIA revealed that the level of AURKA, BIRC5, BUB1, CCNB2, CENPA, CENPN, DLGAP5, ECT2, and RFC4 were positively correlated with the expression of hnRNPR. (**D**) GEPIA indicated that the expression of AURKA, BIRC5, BUB1, CCNB2, CENPA, CENPN, DLGAP5, ECT2, and RFC4 was upregulated in TCGA STAD. (**D**) The expression correlation was determined with Pearson's correlation analysis. *, $P<0.05$.
