# Supplementary material for: HnRNPR-CCNB1/CENPF axis contributes to gastric cancer proliferation and metastasis
Source: Aging (Albany NY). 2019 Sep 16;11(18):7473–91. doi: 10.18632/aging.102254 (PMC6782008; doi:10.18632/aging.102254)
Supplement: Supplementary Tables 1-3 [file aging-11-102254-s002.pdf]

SUPPLEMENTARY TABLES

Supplementary Table 1. Sequences of primers used for qRT-PCR in this study.

Name		Sequences
HNRNPR	Forward (5′-3′)	ATTCCAAGCGTCGTCAGACCAAC
	Reverse (5′-3′)	AATAGTCACCACCTTGCTGAAGCG
CCNB1	Forward (5′-3′)	CTTGCAGTAAATGATGTGGATG
	Reverse (5′-3′)	GTGACTTCCCGACCCAGTAG
CENPF	Forward (5′-3′)	AAAGAAACAGACGGAACAACTG
	Reverse (5′-3′)	CCAAGCAAAGACCGAGAACT
GAPDH	Forward (5′-3′)	CTTAGTTGCGTTACACCCTTTCTTG
	Reverse (5′-3′)	CTGTCACCTTCACCGTTCCAGTTT

Supplementary Table 2. Primary antibodies used in this study.

Antigens	Manufacturer	Application
hnRNPR	Abcam (ab30930)	1:1000 for WB, IHC
CCNB1	Abcam (ab72)	1:1000 for WB, 1:400 for IHC
CENPF	Abcam (ab5)	1:1000 for WB
Ki67	Abcam (ab15580)	1:500 for IHC
MMP9	Abcam (ab38898)	1:500 for IHC
GAPDH	Cell Signaling Tech(D4C6R)	1:1000 for WB

Supplementary Table 3. hnRNPR target cell cycle pathway based on GSEA.

Cell cycle pathway	G1 pathway	G2 pathway	Overlapped by two groups
CCNB1	CDC25A	CHEK1	CDC25A
CDC25A	CDK2	CCNB1	CDK2
RBL1	HDAC1	CDC25A	CDK1
CDK2	DHFR	CHEK2	TFDP1
CDK1	CDK1	BRCA1	E2F1
TFDP1	TFDP1	CDC25C	CCNE1
CDK7	SKP2	CDK1	RB1
CCNH	E2F1	PLK1	CCNB1
E2F1	CCNE1	PRKDC	
CCNE1	RB1	YWHAQ	
RB1	GSK3B	RPS6KA1	
	TP53	CDC25B	
	CDKN1B	YWHAH	
	CDK4	WEE1	
	ATR		
	CCND1		
	CDK6		

Please browse Full Text version to see the data of Supplementary Table 4.

Supplementary Table 4. hnRNPR target tumor metastasis based on GSEA.
