# Supplementary material for: HnRNPR-CCNB1/CENPF axis contributes to gastric cancer proliferation and metastasis
Source: Aging (Albany NY). 2019 Sep 16;11(18):7473–91. doi: 10.18632/aging.102254 (PMC6782008; doi:10.18632/aging.102254)
Supplement: Supplementary Table 4 [file aging-11-102254-s001.docx]

**Supplementary Table 4. HnRNPR target cell cycle pathway based on GSEA.**

| BIDUS_METASTASIS_UP | WINNEPENNINCKX_MELANOMA_METASTASIS | VANTVEER_BREAST_CANCER_METASTASIS | Overlapped | |
| --- | --- | --- | --- | --- |
| HNRNPR | BUB1 | BUB1 | BUB1 |  |
| SRSF1 | TYMS | NUSAP1 | DLGAP5 |  |
| BUB1 | NCAPH | MAD2L1 | CCNB2 |  |
| NUSAP1 | NCAPG | EZH2 | CENPF |  |
| CCNA2 | RFC5 | MCM6 | CENPA |  |
| TYMS | RAD54L | FBXO5 | RFC4 |  |
| NCAPH | MCM6 | ORC6 | ECT2 |  |
| PLK4 | KIF2C | DLGAP5 | CENPN |  |
| RFC5 | CHEK1 | DEPDC1 | BIRC5 |  |
| MAD2L1 | RRM2 | HJURP | AURKA |  |
| LMNB1 | CCNB1 | RAD21 |  | |
| KHDRBS1 | EXO1 | DTL |  | |
| CHEK1 | CDCA5 | SYNCRIP |  | |
| RRM2 | SRSF10 | NDC80 |  | |
| CCNB1 | KIF11 | CCNB2 |  | |
| RRM1 | DLGAP5 | ASPM |  | |
| TTF2 | RACGAP1 | STMN1 |  | |
| MCM10 | PAICS | ATAD2 |  | |
| KIF11 | SMC2 | KIF14 |  | |
| CASC5 | NCAPG2 | CENPF |  | |
| WDHD1 | NASP | SPC25 |  | |
| DLGAP5 | NDC80 | SKA3 |  | |
| RACGAP1 | NUDCD1 | MELK |  | |
| RBBP4 | CDC45 | DIAPH3 |  | |
| DEPDC1 | DSCC1 | CCNE2 |  | |
| MRPL42 | CCNB2 | PRC1 |  | |
| TARDBP | ASPM | UCHL5 |  | |
| CCNB2 | NEIL3 | CENPA |  | |
| PBK | SGOL2 | DCK |  | |
| PTGES3 | MCM4 | RFC4 |  | |
| KIF4A | CDCA8 | TRIP13 |  | |
| WHSC1 | ATAD2 | GPSM2 |  | |
| CENPF | UBE2T | INTS7 |  | |
| HNRNPK | WHSC1 | SMC4 |  | |
| SFPQ | XPO1 | ECT2 |  | |
| SSRP1 | CENPF | PTDSS1 |  | |
| TPX2 | PCNA | GMPS |  | |
| SRSF2 | SPC25 | CENPN |  | |
| H2AFZ | MELK | MRPL13 |  | |
| TSR1 | HSPD1 | BIRC5 |  | |
| CDC23 | RBMX | PAQR3 |  | |
| HNRNPU | CCT4 | FAM64A |  | |
| DKC1 | PRIM2 | HMGB3 |  | |
| ERCC6L | SPAG5 | QSOX2 |  | |
| HNRNPA2B1 | TIMELESS | ARMC1 |  | |
| WBP11 | GINS2 | PGK1 |  | |
| CSE1L | ZNF367 | GPR180 |  | |
| CENPA | KNTC1 | AURKA |  | |
| SMC1A | NUF2 | TFRC |  | |
| EIF4E | PRC1 | CKS2 |  | |
| TUBGCP4 | GPN3 | STK3 |  | |
| FANCI | DDX18 | PSMD7 |  | |
| MPHOSPH9 | ZWINT | AGFG1 |  | |
| RFC4 | CENPA | GBE1 |  | |
| DDX21 | PSMC3IP | MTDH |  | |
| FEN1 | NEK2 | ZDHHC20 |  | |
| LARP4 | KPNA2 | MLLT10 |  | |
| G3BP1 | RANBP1 | CDC25B |  | |
| SMC4 | CEP55 | MTMR2 |  | |
| ECT2 | KIF18B | TK1 |  | |
| DHFR | UHRF1 | SLC7A1 |  | |
| CENPN | DONSON | MGAT4A |  | |
| GMNN | RFC4 | IVNS1ABP |  | |
| CDK1 | OLA1 | NIPA1 |  | |
| ASF1B | CCT7 |  |  | |
| EIF4A3 | NCBP1 |  |  | |
| PATL1 | CCT5 |  |  | |
| NUP153 | CACYBP |  |  | |
| BIRC5 | CHORDC1 |  |  | |
| RRN3 | SHCBP1 |  |  | |
| CKAP5 | ANLN |  |  | |
| TMEM33 | PWP1 |  |  | |
| TMEM194A | MSH6 |  |  | |
| YWHAE | ECT2 |  |  | |
| TFDP1 | DHFR |  |  | |
| PRKDC | GMPS |  |  | |
| AMD1 | CENPN |  |  | |
| HNRNPD | GMNN |  |  | |
| HBS1L | CDK1 |  |  | |
| NRAS | CDKN3 |  |  | |
| CBLL1 | BIRC5 |  |  | |
| RABGGTB | KIAA0101 |  |  | |
| TRA2B | PTTG1 |  |  | |
| SMS | HSPA4 |  |  | |
| DNAAF2 | IMMT |  |  | |
| VBP1 | TARS |  |  | |
| AZIN1 | IWS1 |  |  | |
| ODC1 | CKS1B |  |  | |
| CEP57 | SMARCA5 |  |  | |
| HMGB3 | TAF1A |  |  | |
| CBFB | TCOF1 |  |  | |
| RCOR1 | AURKA |  |  | |
| ADNP | MRPS5 |  |  | |
| MED14 | CKS2 |  |  | |
| KCTD6 | DTYMK |  |  | |
| HNRNPH3 | DNAJA1 |  |  | |
| HMGXB4 | C9orf40 |  |  | |
| PSMD10 | SKA2 |  |  | |
| UBE2C | IPO7 |  |  | |
| NONO | NME1 |  |  | |
| ELAVL1 | GLRX2 |  |  | |
| CCDC15 | ENY2 |  |  | |
| RAB28 | CHTOP |  |  | |
| AURKA | SNRPG |  |  | |
| C5orf22 | MRPL32 |  |  | |
| TFRC |  |  |  | |
| VDAC1 |  |  |  | |
| YBX1 |  |  |  | |
| GAS2L3 |  |  |  | |
| NDRG3 |  |  |  | |
| RNF138 |  |  |  | |
| BCL7A |  |  |  | |
| H2AFY |  |  |  | |
| HSP90AA1 |  |  |  | |
| UBXN2B |  |  |  | |
| CCDC47 |  |  |  | |
| SRSF9 |  |  |  | |
| P4HA1 |  |  |  | |
| WAC |  |  |  | |
| MAPKAPK5 |  |  |  | |
| MTDH |  |  |  | |
| RAE1 |  |  |  | |
| NAP1L1 |  |  |  | |
| DBF4 |  |  |  | |
| HSF2 |  |  |  | |
| MAPK1 |  |  |  | |
| AZI2 |  |  |  | |
| MLLT10 |  |  |  | |
| PPP2CA |  |  |  | |
| CEBPG |  |  |  | |
